# Advancing Simulation-Based Orthopaedic Surgical Skills Training: An Analysis of the Challenges to Implementation

**DOI:** 10.1155/2019/2586034

**Published:** 2019-09-02

**Authors:** Kivanc Atesok, Shepard Hurwitz, Donald D. Anderson, Richard Satava, Geb W. Thomas, Ted Tufescu, Michael J. Heffernan, Efstathios Papavassiliou, Steven Theiss, J. Lawrence Marsh

**Affiliations:** ^1^Harvard University, Beth Israel Deaconess Medical Center, Department of Neurosurgery, Boston, MA, USA; ^2^University of North Carolina, Department of Orthopaedic Surgery, Chapel Hill, NC, USA; ^3^University of Iowa, Department of Orthopaedics and Rehabilitation, Iowa City, IA, USA; ^4^University of Washington, Department of Surgery, Seattle, WA, USA; ^5^University of Manitoba, Section of Orthopaedic Surgery, Winnipeg, MB, Canada; ^6^Children's Hospital of New Orleans, LSU Health Science Center, Department of Orthopaedic Surgery, New Orleans, LA, USA; ^7^University of Alabama at Birmingham, Department of Orthopaedic Surgery, Birmingham, AL, USA

## Abstract

Simulation-based surgical skills training is recognized as a valuable method to improve trainees' performance and broadly perceived as essential for the establishment of a comprehensive curriculum in surgical education. However, there needs to be improvement in several areas for meaningful integration of simulation into surgical education. The purpose of this focused review is to summarize the obstacles to a comprehensive integration of simulation-based surgical skills training into surgical education and board certification and suggest potential solutions for those obstacles. First and foremost, validated simulators need to be rigorously assessed to ensure their feasibility and cost-effectiveness. All simulation-based courses should include clear objectives and outcome measures (with metrics) for the skills to be practiced by trainees. Furthermore, these courses should address a wide range of issues, including assessment of trainees' problem-solving and decision-making abilities and remediation of poor performance. Finally, which simulation-based surgical skills courses will become a standard part of the curriculum across training programs and which will be of value in board certification should be precisely defined. Sufficient progress in these areas will prevent excessive development of training and assessment tools with duplicative effort and large variability in quality.

## 1. Introduction

Simulation-based surgical skills training is widely recognized as a valuable method for improving trainees' performance and as an essential part of a comprehensive curriculum in surgical education [1–6]. Despite tremendous efforts to integrate simulation into orthopaedic surgical education, there is a continued need for simulation-based education to be a more substantial part of orthopaedic curricula. Rapidly growing orthopaedic techniques along with increasing subspecialization necessitate the wide and efficacious adaptation of simulators as basic educational tools in orthopaedic curricula. Evidence suggests that surgical simulation has positive effects on improving trainees' performance in the operating room (OR) [7–10]. As a result, there is currently a consensus among surgical specialties including the orthopaedic community that ideally the development of trainees' surgical skills should commence in a simulated training environment prior to progression to the OR. The purpose of the current review is to summarize the obstacles to, and provide recommendations for, a comprehensive integration of simulation-based surgical skills training into surgical education and board certification.

## 2. Overcoming Barriers to the Integration of Simulation-Based Surgical Skills Training into Surgical Education

### 2.1. Validation of Simulation-Based Surgical Skills Training

Although trainees from all levels—residents, fellows, and practicing surgeons—appreciate the introduction of simulation into surgical education, validation of simulation-based surgical skills training is the most important area that still needs further improvement. Simulators and curricula must be rigorously assessed and validated so that training can be proven to be valuable and performance can be reliably assessed.

Simulated surgical skills training should pass multiple validity tests prior to routine use in surgical curricula and competency assessment. Standard validity tests include face, content, construct, and concurrent validity. In the following paragraphs, we will define and elucidate the subtle differences between these key validity tests.

*Face validity* refers to a simulator's relevance as it appears to participants. In other words, a simulator platform can be said to have face validity if it “looks like” it correctly simulates the intended task/situation. Face validity is usually assessed subjectively via participants' responses to postsimulation questionnaires. Arikatla et al. assessed the face validity of a virtual navigation task trainer developed to simulate laparoscopic surgery tasks. After performing the tasks, the participants were asked to rate the simulator's face validity from 1 (not realistic/useful) to 5 (very realistic/useful) on a 10-item questionnaire. The subjects rated the simulator as highly useful by responding to 90% (9 out of 10) of the questions with a score of three or above [11].

*Content validity* refers to the extent to which a simulator measures the relevant aspects of the surgical task under study. For example, a simulator may lack content validity if it only assesses the accuracy of the reduction and hardware used in a simulated fracture fixation but fails to account for the correctness in the stepwise performance of the procedure. Alsalamah et al. studied the content validity of a virtual reality simulator reflecting real transvaginal ultrasound (TVUS) scanning [12]. The content validity assessment statements aimed to include all the aspects of TVUS scanning, such as the simulator's ability to test normal gynaecological anatomy and early pregnancy structures, and also the simulator's realism in providing measurements of endometrial thickness, ovaries, and crown-rump length. Participants scored the simulator on a visual analog scale based on their subjective perceptions of the simulator's accuracy in assessing the items included in the content validity scoring list. The median scores demonstrated strong agreement with the content validity statements.

*Construct validity* defines the degree to which the simulator measures the specific surgical skills that it was designed for. Construct validity can be described as the appropriateness of inferences made on the basis of test scores. In surgical simulation research, experience levels of subjects testing on a simulator are often used to asses construct validity. For example, if a simulator correctly detects expected variations in the proficiency levels between the expert and novice subjects—in other words, if it correctly identifies quantifiable aspects of a surgical skill—construct validity has been achieved. Lopez et al. studied the construct validity of an arthroscopy simulator in a group of participants that included medical students, residents, and attending physicians [13]. The arthroscopic surgical simulator objectively demonstrated that the attending physicians and senior residents performed at a higher level than the junior residents and novice medical students.

*Concurrent validity* refers to the correspondence between trainees' performance on a tested simulator and a previously validated measure. Researchers often assess the concurrent validity of surgical simulators by comparing participants' simulator scores with their objective structured assessment of technical skill (OSATS) scores. However, comparison with another previously validated simulator can also be used to endorse concurrent validity. In a study including three groups of participants with different levels of expertise (novice, intermediate, and expert) in pedicle screw insertion, Fürst et al. [14] assessed the face, content, construct, and concurrent validity of a novel simulator for minimally invasive spine surgery . The authors used questionnaires with a 5-point Likert scale to assess face and content validity. They evaluated the construct validity using a simulation-based performance score that they calculated for pedicle screw insertion and compared among the three groups. To establish concurrent validity, the performance of each participant was assessed by a specialist using a task-specific checklist and OSATS, and the association between the specialist rating and the simulation-based performance score was evaluated. Their results demonstrated significantly better simulation-based performance scores in the expert group (construct validity) compared with the novices (*P*=0.001) and intermediates (*P*=0.01). The association between the specialist's ratings and the simulation-based performance score (concurrent validity) was strong (*R* = 0.86, *P*=0.01).

Although it is essential to prove face, content, construct, and concurrent validity for a simulation-based training platform to be considered for integration into educational curricula, *transferability* of the skills practiced on a simulator to the OR to perform on patients should also be achieved. In a randomized study, Howells et al. investigated the effect of laboratory-based simulator training on surgical trainees' ability to perform diagnostic arthroscopy of the knee in the OR [15]. The simulator-trained group performed significantly better than the control, demonstrating the transferability of psychomotor skills from simulator training to arthroscopy in the OR.

Arguably, the correlation between performance on the simulator with OR performance on patients provides the most convincing evidence to move forward. The reliability of both the simulated measurement of skills and the measures obtained from actual practice along with good-to-excellent correlation between these two assessments is an absolute necessity for a simulation-based surgical skills course to become part of a standardized educational curriculum. Unfortunately, demonstrating transferability of surgical skills requires fairly sophisticated research methodology, a sufficient number of learners to have the power to demonstrate important differences, and patient consent for the operating room portion of the trial. These issues provide substantial challenges to this type of validation of a simulation course.

#### 2.2. Toward Evaluating Surgical Skills in the OR

Evaluating learners' surgical skills in the OR environment after simulation-based training in laboratories requires objective and standard measurement techniques that are also sensitive enough to differentiate various levels of expertise [16]. A fundamental challenge preventing the movement toward competency-based education is establishing objective criteria for when a resident is ready to advance to the next level. It is possible that the main reason for not being able to establish such criteria is the lack of standard, objective, reliable, and sensitive surgical skill measurement methods. Szasz et al. reviewed 6,814 publications and defined 85 surgical simulator studies that assessed trainees' technical competence [17]. The authors noted that “Very few studies identify standard-setting approaches that differentiate competent versus noncompetent performers; subsequently, this has been identified as an area with great research potential.”

Specific surgical skills for various types of procedures can be hard to define, making it even more difficult to precisely measure them. For example, an individual skill such as navigating a surgical wire into a specific location in a bone is a complex interplay of task-specific knowledge, general knowledge of the anatomy and tools, hand-eye coordination, team communication, dexterity, self-control, experience, and a bit of luck. Hence, defining the essential elements (tasks) of the procedure-specific and surgical skills to be measured is critically important for accurate assessments of competency in the OR.

By definition, all procedures are comprised of specific tasks, and all tasks are comprised of specific skills. The most common method is to perform “task deconstruction,” which accurately defines each task (e.g., anastomosis) and breaks that task into each of its elemental skills (e.g., tissue alignment, needle driving, suturing, and knot tying). It is true that subspecialties need to develop skills that are unique to their specialty; however, basic skills such as suturing, knot tying, developing dissection planes, and anastomosis are truly applicable to all orthopaedic (and other surgical) subspecialities as well.

**Figure 1:**
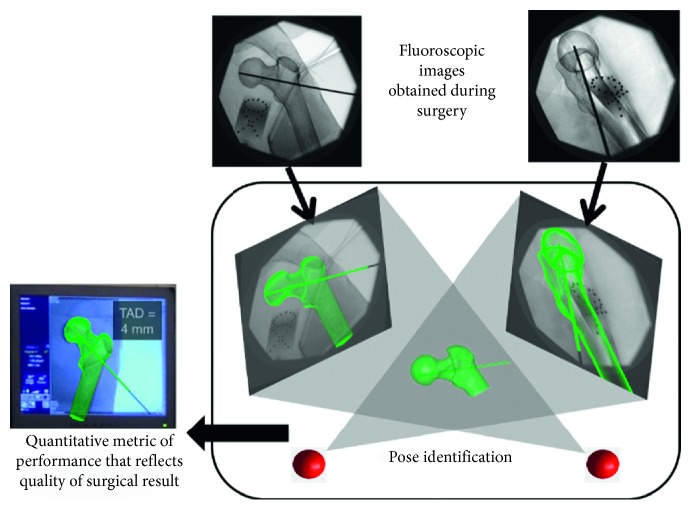
Image analysis software can be used to quantify the metrics for the quality of the surgical result (courtesy of Donald Anderson from the University of Iowa, Department of Orthopedics and Rehabilitation, Iowa City, IA, USA).

One other, more global approach to assessing surgical skills in a clinical setting is to systematically observe the objective elements of a surgeon's behavior. Currently, surgical task-specific checklists and OSATS are two commonly used techniques for measuring surgical skills; however, studies demonstrated that OSATS scores do not always correlate well with the outcomes [18, 19]. In a study of simulated distal radius fracture fixation, Putnam et al. found that the participants' OSATS scores had no correlation with the actual mechanical strength of the fixation they performed [19]. Therefore, to be able to tie simulation-based surgical skills training to OR performance and surgical results, meticulously defined procedure-specific tasks should be assessed using standard measurement protocols that combine more than one objective technique, such as OSATS, motion analysis, and direct objective metrics, such as the accuracy of reduction, time to skill completion, and strength of a fracture fixation construct (Figure 1) [16].

#### 2.3. Proficiency-Based Progression for Surgical Skills Training

Proficiency-based progression (PBP) is the next-generation approach to overcoming the challenge of defining the measurable criteria for technical performance proficiency in training with simulators. The outcome measures of technical skill performance in PBP are quantitative metrics, which can unambiguously quantify the performance of the learner. In this approach, benchmarks are defined by the performance scores of the experts who undergo the same course [6, 20]. Training with the simulator continues until students meet the benchmark scores for two consecutive trials, regardless of the number of trials or time it requires to meet proficiency benchmarks. PBP method provides quantitative evidence that the student is proficient: evidence-based medicine requires evidence-based education.

In order to build a standard nationwide curriculum for various specialties, benchmarks using multi-institutional experts can be developed for national-level courses. There are such courses accepted by the corresponding boards and recognized as national “standards” because they meet the requirements of the certifying boards of the specialty. Some examples include advanced trauma and life support (ATLS), fundamentals of laparoscopic surgery (FLS), and fundamentals of endoscopic surgery (FES). Orthopaedic surgery does not yet have a nationwide specialty course that the American Board of Orthopaedic Surgery (ABOS) recognizes as standard.

#### 2.4. Addressing Problem Solving and Surgical Adversity

Most of the effort in teaching learners about surgery is spent on the knowledge base needed to arrive at an accurate diagnosis, applying the guidelines from the literature regarding the indications for a surgical procedure, and the experiential learning of the mechanics of conducting the procedure on a human. However, problem-solving and decision-making are not explicitly taught in orthopaedic surgery education [21]. Learning about problem-solving in surgery is often a matter of having to deal with a problem as it arises during a procedure or in the perioperative timeframe [22].

Although much of what is required for a successful surgical result comprises the knowledge and skills to perform a routine procedure, the ability to deal with adversity is another skill that can be taught during residency. Theoretically, if trainees can be taught to anticipate and handle certain potential problems, solving such problems in real-life situations can become a learned behavior. In addition to using technical skills in such challenging situations, nontechnical skills such as communication, situational awareness, ability to handle high stress, and decision-making can be equally important [23]. Simulators that are equipped with and programmed for certain intraoperative challenges can be beneficial in teaching problem-solving and decision-making skills. Currently, there is a need for high-fidelity models that can add intraoperative challenges to the routine performance of a procedure, such as excessive bleeding, dural tears, and iatrogenic fractures.

#### 2.5. Remediation of Poor Performance

Another important aspect of simulation-based training is the remediation of poor surgical skills. Those trainees who perform poorly in the laboratory setting should be entered into a remediation program that includes more time and supervision in the skills lab and personal input from supervising faculty. The orthopaedic literature lacks studies that outline the remediation of struggling trainees. In a report summarizing the Council of Emergency Medicine Residency Directors' (CORD) recommendations for resident remediation, Katz et al. indicated that “Early identification of poor performance is key to successful remediation [24].” In the context of simulation-based orthopaedic skills training, early detection of poor performance can only be achieved by using validated simulators and objective, reliable, and sensitive measurement techniques. Gas et al. [25] tested general surgery residents' knowledge and surgical skills in an objective structured clinical examination-style assessment. Participants who scored poorly at any given task were required to remediate those tasks by practicing the simulated stations with staff surgeons and by using online instructional videos. Residents that required remediation were evaluated again in person by an instructor or sent in self-made videos of their performance that was then graded by an instructor.

#### 2.6. Choosing the Right Simulator

There are three important criteria that educators should consider when choosing a simulator for surgical skills training: fidelity, cost, and feasibility.

Fidelity is defined as “the degree of association, resemblance, or correspondence with the original; exactness” [26]. In other words, fidelity does not define the “prettiness” or “genuineness” of the images or the model. In simulation for technical skills, which are the psychomotor skills and tasks that a learner must acquire, fidelity is about the motions that the learner must perform. The hand motions in the simulation should match the complexity of the motions in the corresponding real procedure. Although fidelity is an important criterion in choosing a simulator in health care, there is not enough literature to prove that high-fidelity simulators are better in terms of achieving skill proficiency or transfer of learned skills to the OR [27–30]. In a prospective randomized crossover study, Tan et al. [27] investigated whether there were any differences in the learning outcomes of participants who had trained to proficiency on low- or high-filaparoscopic surgical simulators. The participants were randomized to either high-fidelity (LapSim, Surgical Science) or low-fidelity (FLS, SAGES) laparoscopic simulators and trained to proficiency in a defined number of tasks. They then crossed over to the other fidelity simulator and were tested. The results demonstrated similar increases in participant scores from the baseline for both the high- and low-fidelity groups. However, FLS-trained participants demonstrated a greater ability to translate their skills to successfully complete LapSim tasks. The authors concluded “… the FLS simulator is superior to the LapSim when teaching basic laparoscopic skills to participants.” In a review article, Norman et al. [28] compared learning from high-fidelity simulation with learning from low-fidelity simulation based on measures of clinical performance. Although both high- and low-fidelity simulation learning resulted in consistent improvements in performance in comparison with no-simulation control groups, nearly all the studies showed no significant advantage of high-fidelity over low-fidelity simulation, with average differences ranging from 1 to 2%.

Complex high-fidelity simulators may cost up to 80,000–90,000 United States Dollars (USD). With further expenses such as maintenance and software upgrades, simulator-based training can become very costly. Although the ultimate goal of simulation-based training is to enhance learning, if the cost associated with implementing effective simulators is prohibitively high, it may not be a viable option [31]. Despite the consistent focus on the high costs of simulation, the literature does not include sufficient research regarding simulators' cost-effectiveness [31]. Berg et al. demonstrated that startup costs and operational expenses of a surgical skills laboratory decreased from 1,151 to 982 USD per resident over a three-year period [32]. It is sensible to assume that the initial expenses of building a simulation-based surgical skills laboratory will be a lot higher compared with the expenses of an established laboratory. Moreover, surgical skills training before residents enter the OR may bring additional savings of increased OR turnover in teaching hospitals.

Choosing the best simulator also depends upon the procedure or specific task that needs to be trained. Simulating full procedures is usually more difficult because the surrogates used for full procedure simulations, such as cadavers and live animal models, are not very feasible options due to procurement, cost, and logistics. Partial manikins with replaceable organs or tissues can be used repeatedly and are more feasible and cost-effective for training of specific simple tasks such as central line placements [33]. Virtual reality (VR) simulators, such as laparoscopic or arthroscopic simulators, have been developed with the addition of haptic properties and replica legs for increased fidelity. These VR simulators are very feasible due to their easy accessibility for trainees, the small space they occupy, the possibility to train with them repeatedly, and their resemblance to real laparoscopic or arthroscopic procedures. However, the cost is still considered as a drawback, especially for high-fidelity haptic arthroscopy simulators [6]. When considering the feasibility of implementing simulator-based training into a surgical educational curriculum, time spent in education and thus away from clinical practice should also be taken into account. Although more simulation-based surgical skills training may result in better learning for residents, conducting lab-based training for extended periods, especially early on in a residency, might not be feasible. As a generalization, the measure of value for a simulator should be as follows: “the fidelity of a simulator should match the complexity of the task or procedure to be trained and/or assessed.” Simple models should be used for basic skills training and more sophisticated computer-based simulators for complex procedures.

## 3. Skills Assessment for Board Certification: What Are the Issues and Areas That Need to Be Improved?

Certifying boards' mission is to set standards for education and practice. Although many competencies are required to meet these standards, for surgical boards, technical skills in performing procedures is a particularly important competency. Since assessing technical skills in the OR is challenging, simulation should play an important role in assessing a skill during the training and certification process. Surgical boards have the opportunity to advance simulation by requiring its use during the steps toward initial and ongoing certification. Some surgical boards have taken initiatives to incorporate simulation into their certification process, but others have encountered barriers that have prevented the wide adoption of board-required simulation-based surgical skills training. In addition to the conservative attitude of opposing any new technology before there are sufficient evidence and validation to prove its efficacy, value, and utility, there is also the natural resistance to change, and the threat and fear of those who risk adverse consequences from the new change. From a practical aspect, there is the issue below of difficulty of transfer from simulation to the OR in order to provide the unequivocal evidence necessary to convince the boards of the value of simulation. Although the ABOS has required simulation-based training as part of residency in postgraduate year one since 2013, the standards of such training and of residents' performance have not been well defined.

One of the challenges for boards implementing simulation-based skills training is the fundamental need for both the simulation course and associated assessments to be rigorously validated prior to implementation. Unfortunately, there is not sufficient high-level evidence in orthopaedic surgery to prove that simulated skills training courses improve performance in the OR or that the associated assessments are reliable and valid. Another important point is that the surgical outcome is not completely determined by the success of the surgical procedure; rather, there are many confounding factors such as whether the surgeon has chosen the right treatment (or procedure) for the patient, the preoperative health of the patient, appropriate prepping before entering the OR, postoperative nonsurgical complications, and patient compliance with postoperative instructions. However, the closest evidence would be, subsequent to training to proficiency on a simulator, if the learner is able to demonstrate proficiency in an animal model that is nearly identical with a given patient procedure. The ABOS has recently sponsored grants through the Orthopaedic Research and Education Foundation to assist investigators in obtaining high‐level evidence on certain simulation-based surgical skills training courses. These grants hold promise for the future of simulation in orthopaedic surgery.

Unfortunately, challenges in simulation-based skills training in orthopaedic surgery are not limited to a lack of sufficient evidence. Even when high-level scientific evidence proves the value of simulation in orthopaedic surgery, implementing simulation-based skills training courses across residency programs can be very expensive. Furthermore, a standard simulation-based skills training course and the associated assessments the board requires must be available locally or at regional testing centers—the logistics of building such a network can be extremely challenging.

## 4. Summary

Simulation-based skills training is becoming increasingly integrated into surgical education as an important teaching method across surgical specialties. There are challenges waiting to be addressed prior to further implementation of simulation in residency curriculum and board certification. These challenges can be summarized as the validation of simulation-based courses; proving that simulation-based surgical training improves trainees' performance in the OR; addressing the issues of expense, fidelity, feasibility, and standardizing assessment techniques; and boards' involvement in implementing simulation as a requirement for board eligibility or certification. Advancing simulation-based skills training will accelerate after the recognition and systematic solution of the challenges outlined in this article.
